# Purified PTEN-Long Induces Liver Cancer Cells to Undergo Autophagy and Apoptosis

**DOI:** 10.3389/fsurg.2022.767611

**Published:** 2022-05-11

**Authors:** Lin Tan, Zeping Xu, Qiqi Mao, Shaocheng Zhou, Jie Zhu, Xie Zhang, Hong Li

**Affiliations:** ^1^Department of Hepatobiliary Surgery, The Affiliated Hospital of Ningbo University, LiHuiLi Hospital, Ningbo, China; ^2^Department of Gastrointestinal Surgery, The Affiliated Hospital of Ningbo University, Ningbo First Hospital, Ningbo, China; ^3^Department of Pharmacy, The Affiliated Hospital of Ningbo University, LiHuiLi Hospital, Ningbo, China

**Keywords:** PTEN-Long, autophagy, apoptosis, liver, cancer

## Abstract

**Background:**

PTEN-Long is a translational variant of phosphatase and tensin homolog deleted on chromosome 10 (PTEN). This tumor suppressor is frequently lost or mutated and even it has been shown as the determinant in several human tumors. Therefore, we will determine the significant roles of PTEN-Long in the development of liver cancer.

**Methods:**

In the present study, we characterized the antitumor effects of PTEN-Long and PTEN in proliferation, migration of HepG2 cells, apoptosis and autophagy in liver cancer cells. To extends, we have also measured the effects of purified PTEN and PTEN-Long in the above index of HepG2 cells.

**Results:**

PTEN and PTEN-Long were ectopic-expressed in HepG2 cells, and their phenotypic effects were recorded. As expected, there was less expression of PTEN-Long and PTEN in liver cancer samples than in paired normal tissues. Ectopic expression of PTEN-Long or PTEN significantly decreased the proliferation and migration of HepG2 cells and increased apoptosis. PTEN ectopic-expression increased the number of GFP-/RFP+-LC3 puncta and levels of beclin-1 and LC3BII/LC3BI, suggesting autophagy induction. Purified PTEN-Long freely entered cells, decreased proliferation, and increased autophagy and apoptosis, while purified PTEN did not.

**Conclusions:**

Our results identify an antitumor function of purified PTEN-Long and suggest its potential utility for liver cancer treatment.

## Introduction

Primary liver cancer is the sixth most common cancer worldwide, and hepatocellular carcinoma (HCC) accounts for 80% of such cancers (1). Data from China suggest that hepatocellular carcinoma's morbidity and mortality rates rank fourth and third among malignant tumors, respectively (2). Surgical resection is effective for the treatment of early-stage liver cancer. Unfortunately, approximately 80% of liver cancer patients already have advanced disease at presentation. For these reasons, liver cancer represents a major therapeutic challenge, and further research focused on the molecular mechanisms of HCC tumorigenesis is essential.

Cell death is a complex process that is carefully regulated. As the first recognized programmed cell death process, the role and regulatory network of apoptosis have gradually become clear nowadays (3). However, apoptosis is not the only factor that determines the fate of cell death. In recent years, autophagy, known as type II programmed cell death process, has been shown to co-regulate cell death with apoptosis. In some cases, autophagy inhibits apoptosis and is a cell survival pathway, but autophagy itself can also induce cell death, or acts together with apoptosis and as a backup mechanism to induce cell death in the case of apoptosis defects. These two pathways are correlated and regulated by each other in different environment (4). The study and utilization of these interactions will be beneficial to further reveal the pathogenesis of liver cancer.

The gene encoding the phosphatase and tensin homolog deleted on chromosome 10 (*PTEN*) is frequently lost or mutated in many late-stage tumors (5). It is the second most prevalent genetic mutation found in several human tumors, including liver cancer. *PTEN* encodes a protein of 403 amino acid residues; it is a tumor suppressor characterized as a dual-specificity protein with both lipid phosphatase and protein phosphatase activities. The protein employs trisphosphate (PIP3) as its primary substrate, which is hydrolyzed to phosphatidylinositol (4, 5)-bisphosphate (PIP2) (6, 7). PTEN blocks the phosphatidylinositol 3-kinase (PI3K) signaling pathway via its lipid phosphatase activity in which PTEN dephosphorylates phosphatidylinositol (3,4,5)-trisphosphate [PI(3,4,5)P3] to form phosphatidylinositol 4,5-bisphosphate [PI(4,5)P2], inhibiting AKT and its downstream signaling pathways; in this manner, the protein inhibits cell growth, proliferation, and survival (8–11). These findings suggest that PTEN represents a critical node in tumor development and might be helpful as a potential therapeutic target in tumor treatment. Nevertheless, its use in clinical trials is limited by the non-secretory nature of PTEN. Its use also presents the risk of the generation of new tumors induced by adenovirus-mediated gene transfer (6).

Recently, two isoforms of PTEN have been identified as translational variants of PTEN, PTEN-Long, and PTENα (7, 12). PTEN-Long is superior to PTEN depending on its specific region, translated from an alternative start site within the 5'-coding region of *PTEN* mRNA. It contains a 173 amino acid-residue domain at its *N* terminus (13). This enhanced region characterizes PTEN-L as a secreted protein, and it is detected in human serum and plasma, unlike the non-secretory protein PTEN (14). Furthermore, the lipid phosphatase and protein phosphatase activities of PTEN-Long are comparable to that of PTEN, and it can display higher activities than PTEN (15). PTEN-Long can be actively secreted from cells and enter other cells, inhibiting PI3K signaling both *in vitro* and *in vivo*. The protein might be involved in the alternatively translated region, including a polyarginine stretch with homology to known cell-permeable peptides (8, 16, 17). Recent studies reported that levels of PTEN-Long are significantly lower in tumor tissues than in normal tissues. Upregulated expression of PTEN-Long inhibits the proliferation of breast cancer and renal cell adenocarcinoma cells and induces tumor regression in murine models of cancer, suggesting that PTEN-Long may serve as a therapeutic target in cancer (13, 18). Nevertheless, the roles of PTEN-Long in the development of liver cancer are unknown.

Here, we study the effects of PTEN-Long on the HCC-derived cell line HepG2. Moreover, we compared the effects of purified PTEN and PTEN-Long on cell migration, apoptosis, and autophagy. Our results demonstrate the potential antitumor activity of purified PTEN-Long, which was not shared by PTEN, suggesting the possible significance of the former as a therapeutic target.

## Materials and Methods

### Tissues

Liver cancer tumor tissues and paired normal tissues (*n* = 25 pairs) were obtained from the affiliated Hospital of Ningbo University, LiHuili Hospital. The LiHuili Hospital Ethics Committee, Ningbo Medical Treatment Center, approved this study. Normal tissues were extracted approximately 1 cm from the tumor margin, and all tissues were immediately stored at −80°C. All diagnoses were histopathologically confirmed as having HCC (Table 1).

**Table 1:** Clinicopathologic features of HCC patients.

		**%**
Age (y)	45–80	
**Sex**		
Male	10	40%
Female	15	60%
Tumor size (cm)	1.6–16	
**Histology**		
HCC	25	100%
**Grade**		
I	13	52%
II	2	8%
IIIA	8	32%
IVA	1	4%
IVB	1	4%
**T classification**		
T1	13	52%
T2	2	8%
T3	9	36%
T4	1	4%
**N classification**		
N0	23	92%
N1	2	8%
**M classification**		
M0	24	96%
M1	1	4%

### Cell Line

HepG2 cells were purchased from the Shanghai Cell Bank, Chinese Academy of Sciences. Cells were cultured in DMEM/high glucose medium (HyClone, Logan, UT, USA) supplemented with 10% fetal bovine serum and 100 U/ml penicillin/streptomycin. Cells were maintained in a humidified incubator.

### Expression Plasmids

The mammalian expression plasmids pcDNA 3.1, pcDNA 3.1-PTEN, and pcDNA 3.1-PTEN-Long were described in a previous study (18). JpExpress404-PTEN-V5/His and JpExpress404-PTEN-Long-V5/His were gifts from Ramon Parsons (Addgene plasmids # 49420 and # 49417).

### Generation of Stable Transfectants

HepG2 cells were transfected in the presence of Lipofectamine 2000 (Life Technology, CA, USA) according to the manufacturer's protocol. Briefly, cells (2 × 10^5^ cells per well) in six-well plates (Nunc, Roskilde, Denmark) were transfected when they reached 80%−90% confluency. Mock transfection with the empty plasmid (pcDNA3.1) served used as a control. The transfection mixtures were diluted in Opti-MEM Reduced Serum Medium (Life Technology), and the HepG2 cells were incubated in this mixture for 6 h. After transfection, the cells were cultured for 2 weeks in 400 μg/mL G418 to generate stable transfectants.

### Western Blot Analysis

Tumor and normal adjacent tissues were crushed with a mortar under liquid nitrogen and suspended on ice in lysis buffer, and the BCA Assay was used (Bio-Rad Laboratories, California, USA) to determine protein concentrations.

Harvested cells (1 × 10^6^) were homogenized in 100 μl RIPA lysis buffer (Solarbio, Beijing, China) supplemented with 1 × protease inhibitor cocktail. Proteins were separated using SDS-PAGE and transferred onto polyvinylidene fluoride membranes (PVDF, Immobilon P; Millipore, Billerica, MA, USA). After blocking in 5% low-fat milk powder in phosphate-buffered saline/Tween-20 (PBST) for 60 min, primary antibodies were incubated with the membranes at 4°C overnight. The antibodies were as follows: anti-PTEN (138G6), anti-AKT (C67E7), anti-p-AKT (Ser473) (D9E), anti-Cleaved caspase 3 (D175), anti-PRAS40 (D23C7), anti-p62/SQSTML (5114S), anti-LC3 (D3U4C), anti-BECLIN-1 (D40C5), anti-Bcl-xl (2764), anti-Bax (2772), anti-p-PRAS40 (Thr246) (D4D2) (Cell Signaling Technology, Danvers, MA, USA), and anti-GAPDH (sc-47724; Santa Cruz Biotechnology, Inc., Dallas, TX, USA). After washing, membranes were incubated with the appropriate secondary antibodies (Santa Cruz Biotechnology) at room temperature for 45 min. Immunocomplexes were visible using an enhanced chemiluminescence detection system. Western blot results were analyzed using ImageJ (NIH).

### Cell Proliferation Assays

MTT [3-(4, 5-dimethylthiazol-2-yl)-2, 5-diphenyltetrazolium bromide] assays were performed according to the manufacturer's protocol (Promega Madison, WI, USA). Briefly, after 2,000 cells per well were seeded into 96-well plates, they were incubated at 37°C for 24, 48, 72, or 96 h. Absorbance was measured at 490 nm using a Microplate Reader (Bio-Rad Laboratories).

### *In vitro* Scratch Assays

Stably transfected HepG2 cells were cultured in 6-well plates and starved for 24 h. A linear scratch wound was introduced into the cell monolayer using a 200-μL pipette tip. The cells were washed with PBS 2–3 times and subsequently cultured in fresh medium without FBS for 72 h. Scratches were observed using an inverted microscope immediately (0 h) and after 72 h.

### Apoptosis Assays

According to the product specification, Apoptosis was measured using a PE Annexin V Apoptosis Detection Kit I (BD, Biosciences, Franklin Lakes, NJ, USA). After labeling with Annexin V, cells (≥ 20,000 cells per sample) were analyzed using flow cytometry (BD Biosciences).

### Autophagic Flux Assays

Cells were infected with the stubRFP-lensGFP-LC3B Lentivirus (19, 20) (GeneChem, Shanghai, China). Images were taken using a confocal microscope 72 h after infection.

### Protein Purification

Proteins were purified from *Escherichia coli* BL21 (DE3; Invitrogen, Carlsbad, CA, USA) transformed by a plasmid encoding JpExpress-PTEN or JpExpress-PTEN-Long. After inductive expression of protein using 0.1 mM isopropyl β-D-1-thiogalactopyranoside (Sigma–Aldrich, St. Louis, MO, USA) for 4.5 h at 21°C, the protein was extracted from bacteria sonicated in lysis buffer (500 mM NaCl, 25 mM Tris, pH 7.5). This was followed by centrifugation at 30,000 × *g* for 30 min, and *E. coli* lysates were filtered through 0.22-μm filters and passed through an AKTA Prime Plus. Proteins were subsequently resolved using SDS-PAGE and quantified using the BCA Assay.

### Statistical Analysis

Experiments were performed at least three times. Each value was expressed as the mean ± standard deviation (SD). Data were analyzed using one-way analysis of variance and a two-sample independent *t*-test SPSS v17.0 (SPSS Inc; Chicago, IL). *P* < 0.05 indicated significant difference.

## Results

### PTEN-Long Expression Is Significantly Reduced in HCC

**Figure 1:**
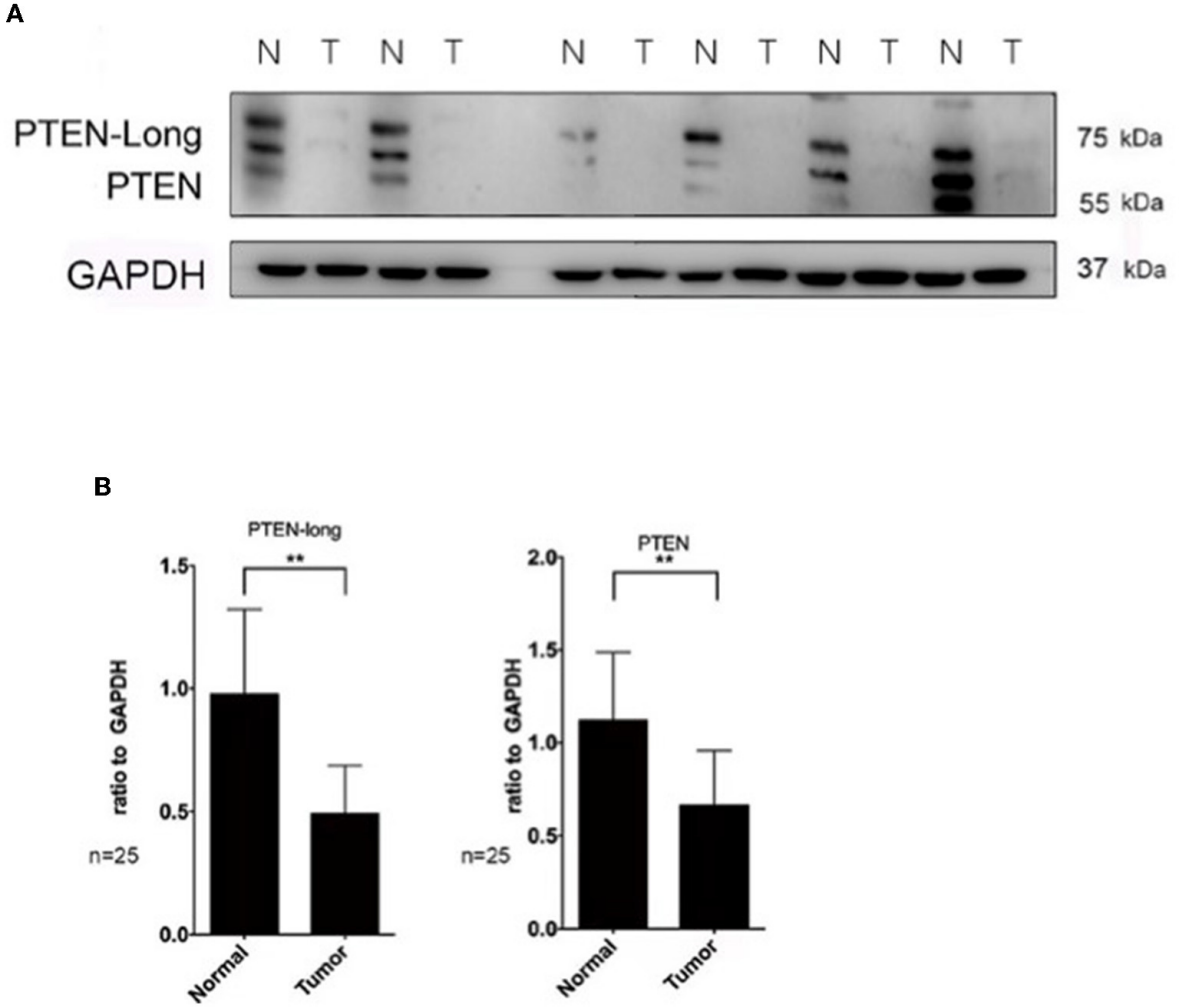
The expression of PTEN-Long is significantly reduced in hepatocellular carcinoma. Western blot analysis of PTEN and PTEN-Long expression in tumor and adjacent normal tissues. **(A)** Immunoblot of six randomly selected PTEN hepatocellular tumors and the corresponding normal tissues (*N* = normal, T = tumor). **(B)** The ratios of PTEN-Long to GAPDH and PTEN to GAPDH in 25 pairs of samples are presented (**P < 0.01).

Western blot analysis revealed that the levels of PTEN-Long and PTEN were reduced by approximately 50% (*P* < 0.01) and 60% (*P* < 0.01) in paired tumor tissues vs. adjacent normal liver tissues (*n* = 25 pairs) (Figures 1A,B).

### PTEN-Long Inhibits the Migration and Invasion of HepG2 Cells

**Figure 2:**
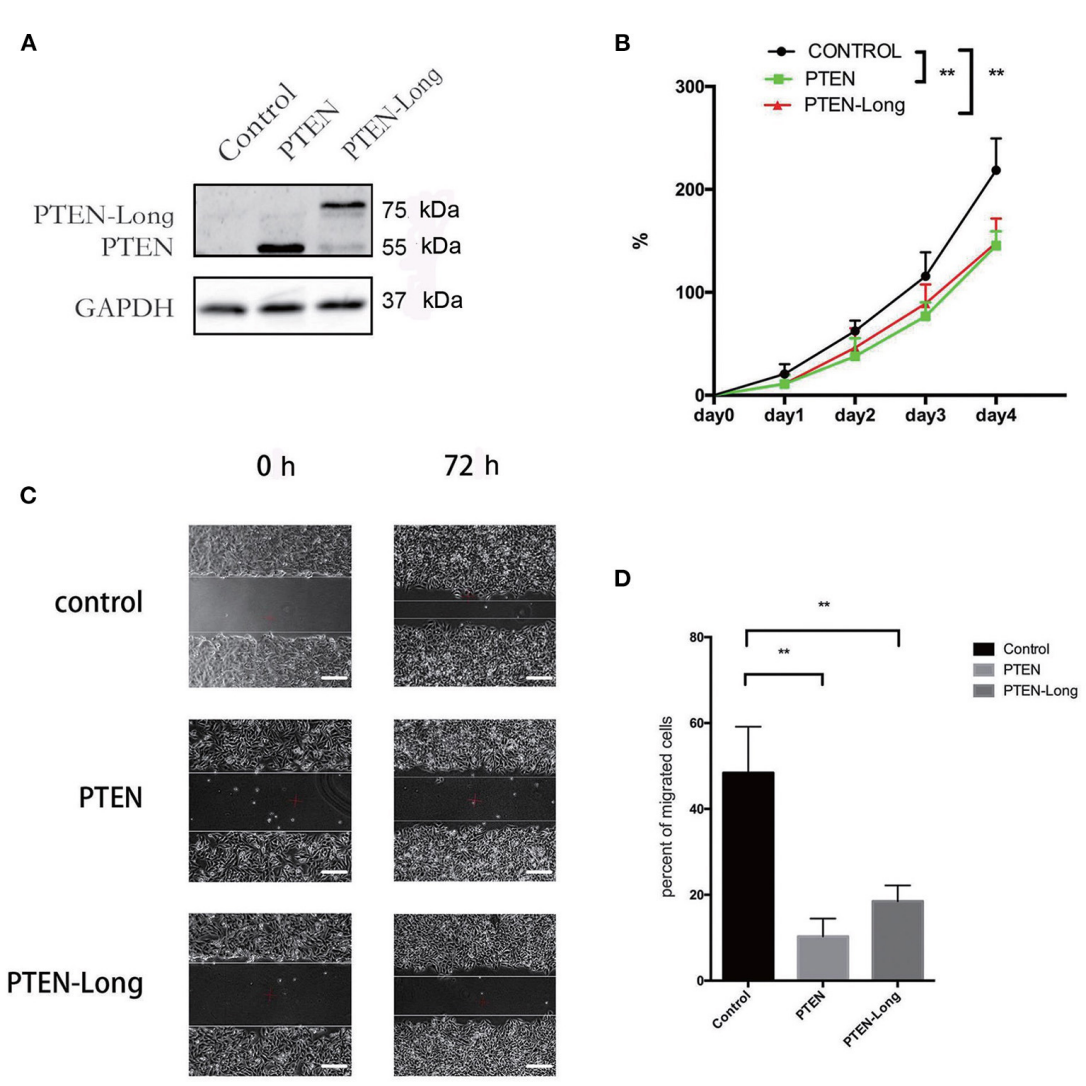
PTEN-Long inhibits cell proliferation and migration. HepG2 cells were selected for at least 2 weeks by incubation with 400 μg/mL G418 after transfection. **(A)** Western blot analysis of lysates prepared from HepG2 cells. **(B)** Of cell proliferation analysis by MTT assay (**P < 0.01). **(C)** Cell migration analysis by scratch assay. Scale bar = 200 μm. **(D)** ImageJ analysis of the invaded area (**P < 0.01).

To investigate the role of PTEN-Long in HCC, HepG2 cells were transfected with pcDNA3.1 plasmids harboring sequences encoding PTEN-Long or PTEN. Western blot analysis revealed ectopic expression of PTEN-Long and PTEN by the transfectants (Figure 2A). The growth rates of the PTEN-Long or PTEN transfectants were significantly different from those of the mock-transfected controls 72 and 96 h after transfection (*P* < 0.01). There was no significant difference between the growth rates of PTEN-Long or PTEN transfectants (Figure 2B). The scratch assay showed that the migration of each PTEN-Long or PTEN transfectant was inhibited compared with those of the controls (Figures 2C,D).

### PTEN-Long Induces HepG2 Cells to Undergo Autophagy

**Figure 3:**
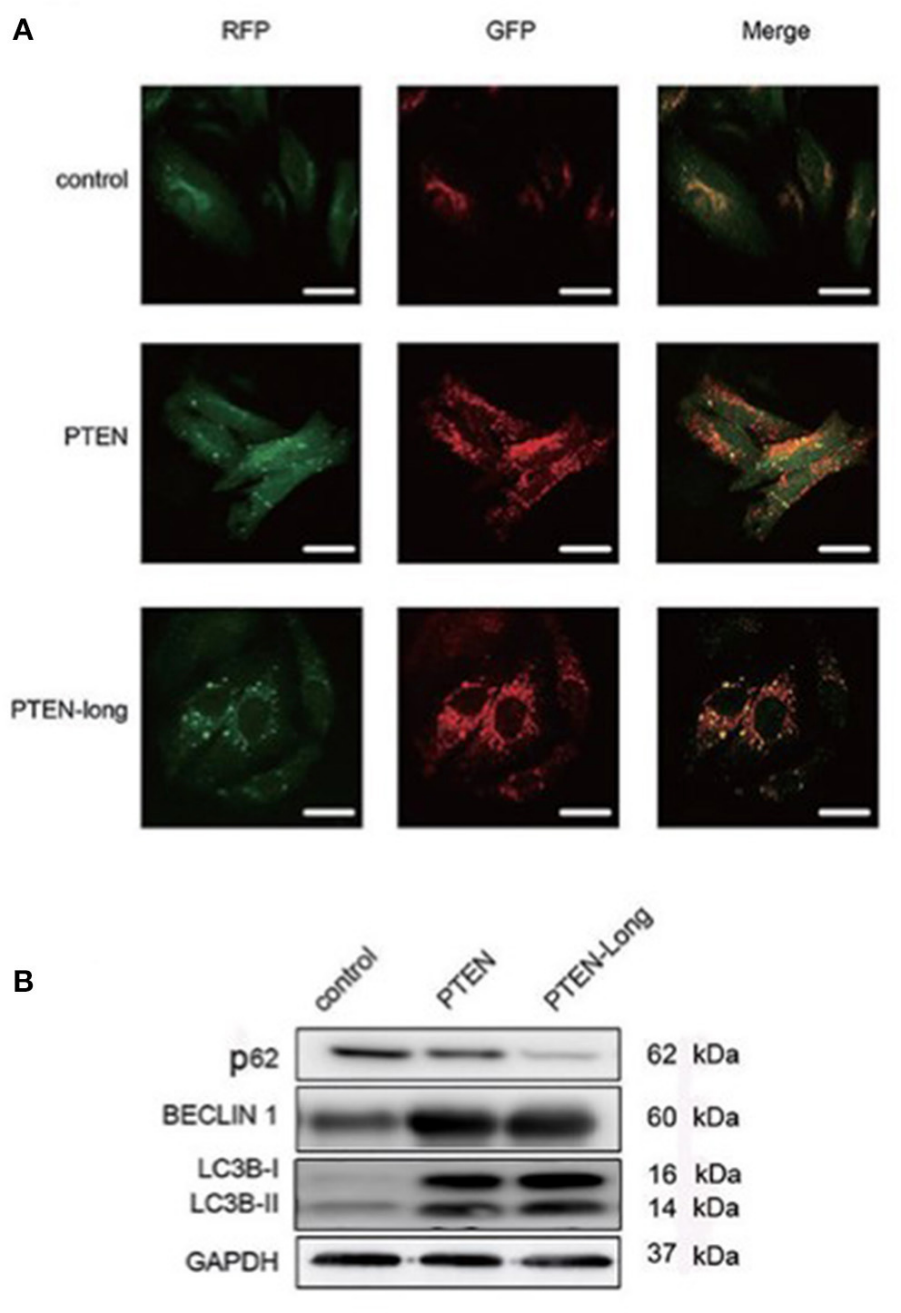
PTEN-Long induces autophagy of HepG2 cells. **(A)** Autophagy analysis of cells ectopic-expressing PTEN or PTEN-Long. Scale bar = 20 μm. **(B)** Western blot analysis of autophagy-related proteins in HepG2 cells.

To study the effect of PTEN-Long on autophagy, cells infected with the stubRFP-lensGFP-LC3B lentiviral vector were subjected to autophagic flux analysis. There were significantly more GFP+/RFP+ puncta in PTEN-Long or PTEN ectopic expressing cells than in controls, suggesting that autophagosomes formed (Figure 3A). Compared with the controls, the PTEN-Long and PTEN ectopic-expressing cells contained more autolysosomes, suggesting autophagy induction. Western blot analysis of the expression of the autophagy-related proteins p62, beclin-1, and LC3BII/I revealed that the expression of p62 was significantly reduced, while the levels of BECLIN-1 and the ratios of LC3BII/LC3BI levels were significantly increased in the PTEN-Long and PTEN transfectants compared with controls, suggesting increased autophagic activity (Figure 3B).

### PTEN-Long Induces HepG2 Cells to Apoptosis

**Figure 4:**
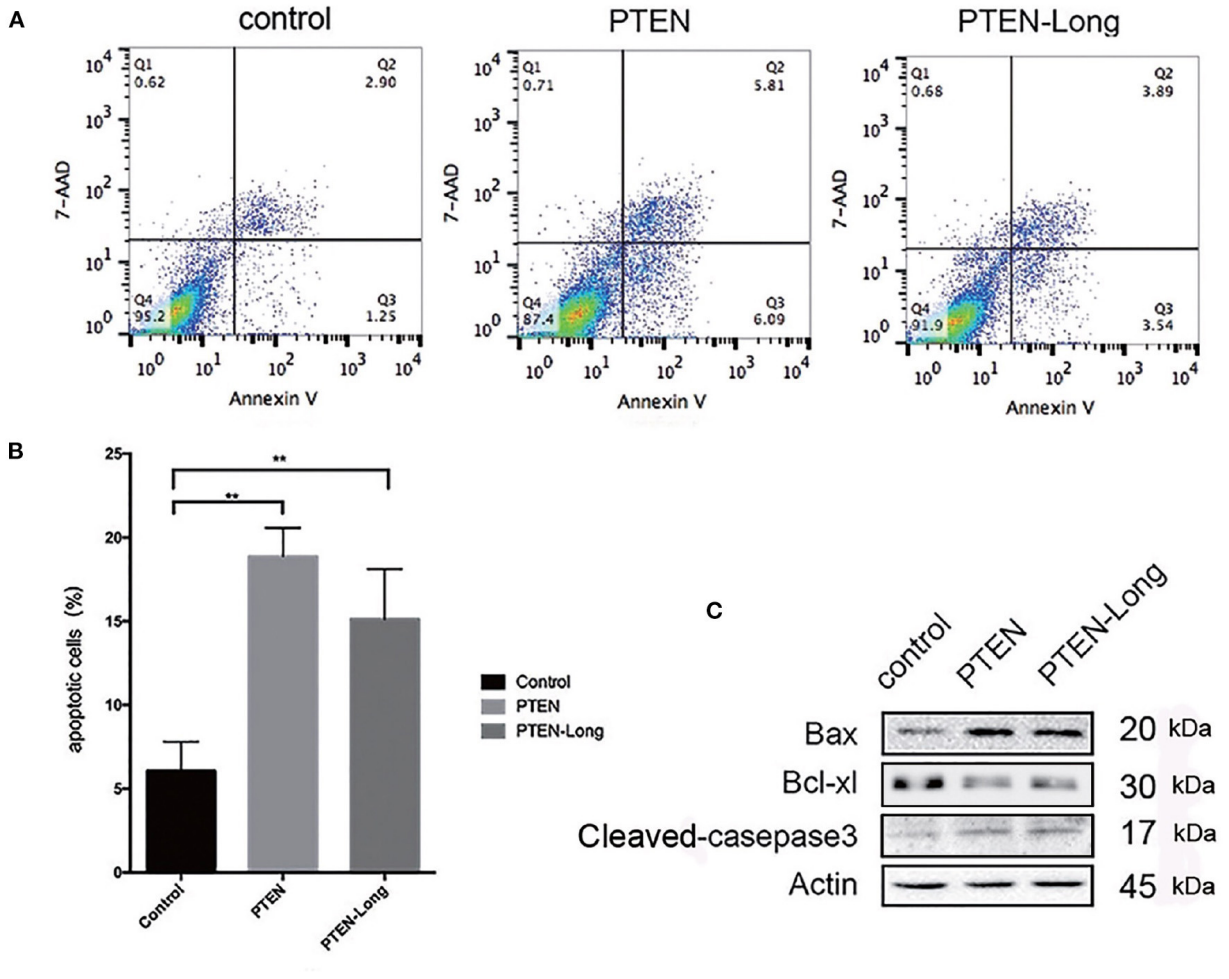
PTEN-Long induces apoptosis of HepG2 cells. **(A)** Flow cytometric analysis of apoptosis. **(B)** The proportion of apoptotic cells (Annexin V+, 7-ADD-) calculated using FlowJo (**P < 0.01). **(C)** Western blot analysis of apoptosis-related proteins in HepG2 cells.

The ectopic expression of PTEN-Long and PTEN induced HepG2 cells to undergo apoptosis (Figure 4A). The proportions of apoptotic cells in cultures of the PTEN and PTEN-Long transfectants were approximately 3-fold higher than cultures of the control cells (Figure 4B). Levels of the apoptosis-related proteins cleaved caspase 3 and Bax were increased, whereas expression level of BCL-XL was decreased compared with those of the control cells (Figure 4C).

### PTEN-Long Suppresses PI3K/AKT Signaling in HepG2 Cells

**Figure 5:**
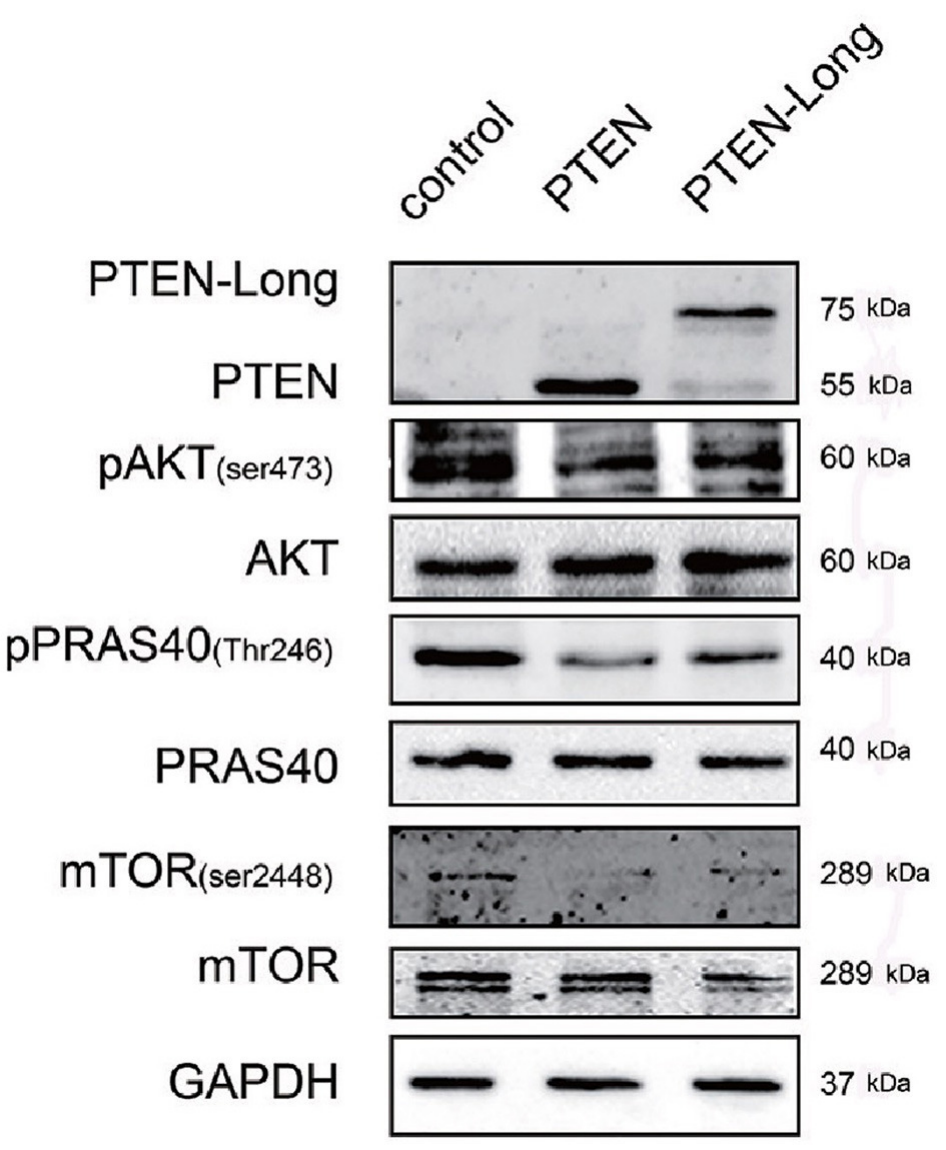
PTEN-Long inhibits PI3K-AKT signaling. Western blot analysis of PI3K-AKT signaling-related proteins in HepG2 cells ectopic-expressing PTEN or PTEN-Long.

PTEN suppresses the classical PI3K/AKT pathway. Therefore, we investigated the effect of PTEN-Long on this pathway in HepG2 cells. The levels of p-AKT (Ser473), p-PRAS40 (Thr246), and p-mTOR (Ser2448) were significantly lower in the PTEN-Long- and PTEN transfectants than in control cells (Figure 5), suggesting the suppression of PI3K/AKT pathway activity. However, there were no significant differences between PI3K/AKT signaling in the PTEN and PTEN-Long transfectants.

### Purified PTEN-Long, but Not PTEN, Inhibits Cell Migration and Induces HepG2 Cells to Undergo Autophagy and Apoptosis

**Figure 6:**
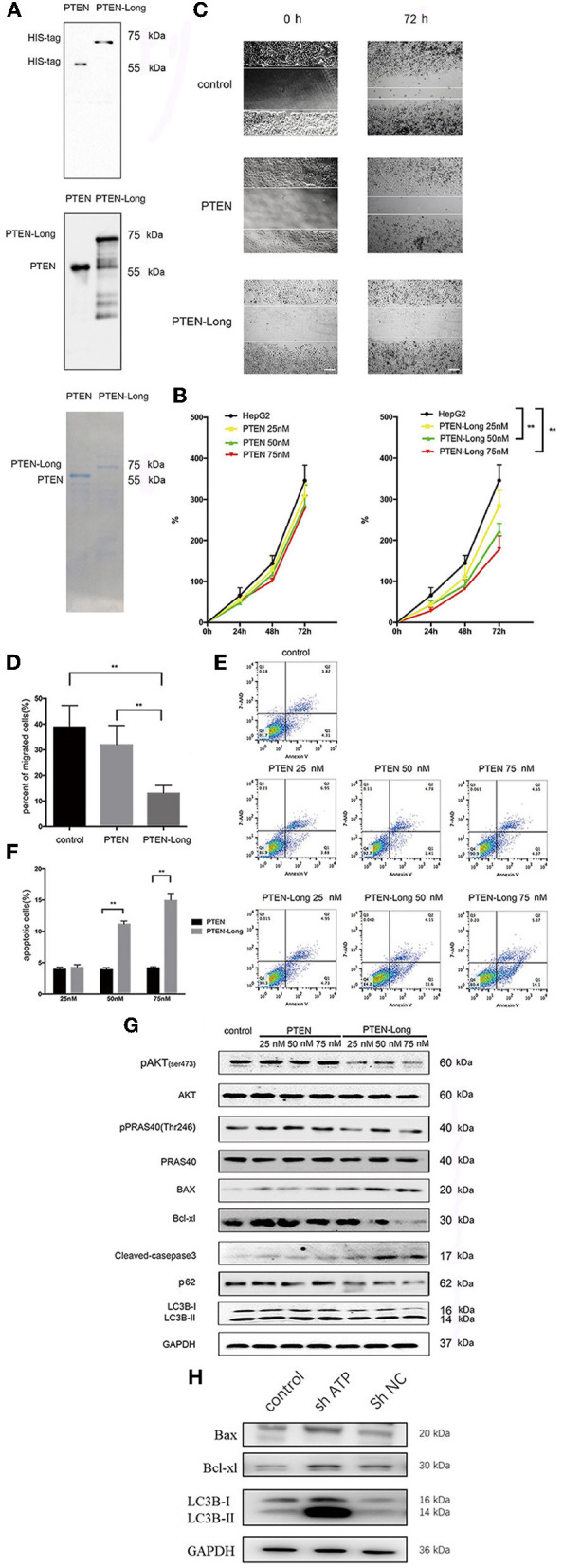
Purified PTEN-Long induces autophagy and apoptosis of HepG2 cells. **(A)** Western blot analysis of purified protein PTEN isoforms. **(B)** Cell proliferation analysis by MTT assay (**P < 0.01). **(C)** Cell migration analysis by scratch assay. Scale bar = 100 μm. **(D)** ImageJ was used to analyze the invaded area indicated in **(C)** (**P < 0.01). E. Flow cytometric analysis of apoptosis. **(F)** The proportions of apoptotic cells (Annexin V+, 7-AAD-) indicated in **(E)** were calculated using FlowJo (**P < 0.01). **(G)** Western blot analysis of protein expression after treatment of HepG2 cells with purified PTEN or PTEN-Long. **(H)** Western blot analysis of autophagy- and apoptosis- relative protein expression after suppressing of AKT expression.

The generation of purified PTEN-Long and PTEN is shown in Figure 6A. PTEN-Long inhibited the growth of HepG2 cells in a concentration-dependent manner, and the most significant difference was detected when cells were treated with 75 nM PTEN-Long, while no effects were observed in cells treated with purified PTEN (Figure 6B). Similarly, treatment with PTEN-Long, not PTEN, significantly inhibited cell migration (Figures 6C,D) and induced apoptosis in a concentration-dependent manner (Figures 6E,F). We detected increased levels of autophagy- and apoptosis-related proteins and inhibition of PI3K/AKT signaling (Figure 6G). At the same, we found that downregulated AKT was negatively regulated with autophagy- and apoptosis-related proteins, which means PTEN-Long might upregulate autophagy and apoptosis process through inhibition of PI3K/AKT signaling (Figure 6H).

## Discussion

Liver cancer is one of the most prevalent cancers worldwide. Surgery is the primary treatment for liver cancer, but it is mainly administered to patients with early-stage disease. Unfortunately, the few available treatments for advanced liver cancer are insufficient. Although chemotherapy has been used for over 30 years to treat liver cancer, definitive evidence of prolonged survival time is lacking (21, 22). Resistance to chemotherapeutic drugs remains a significant barrier and often leads to treatment failure (15). There are more opportunities for targeted therapy with advanced knowledge of liver cancer genetics and liver cancer-related molecular pathways. The PTEN-PI3K-AKT pathway has been a compelling target in the clinical trials of cancer treatment (22). Activation of the PTEN-PI3K-AKT signaling pathway is involved in normal cell proliferation, survival, and migration; however, its abnormal activation promotes cancer cell growth (11). Expression levels of mTOR and its downstream P70S6K in the PI3K-AKT-mTOR signaling pathway are usually upregulated in liver cancer compared to paracancerous and normal liver tissues (20). In this study, we showed that the deficiency of PTEN and increased levels of p-AKT and p-mTOR were associated with tumor grade, intrahepatic metastasis, vascular invasion, TNM stage, and high Ki-67 labeling index in liver cancer or HepG2 cells, emphasizing again that PTEN can act as a tumor suppressor through inhibition of the proliferation and migration of HCC via PTEN-PI3K-AKT pathway.

Despite evidence to suggest that PTEN serves as a potential anti-tumor therapeutic molecule, there are drawbacks associated with using PTEN as a gene target. For example, adenovirus technology can directly increase the levels of PTEN (16, 23); however, this technique has adenovirus-associated side effects (5). Furthermore, PTEN expression can be increased indirectly using microRNAs (16, 24–26). Although microRNAs inhibit tumor growth by increasing PTEN expression, they are not currently used as medications due to their broad off-target effects. Considering the difficulties of delivering a therapeutic vector containing PTEN to target cells in gene therapy, PTEN-Long could be efficiently delivered anywhere via the circulation without any vector.

As the critical role of classical PTEN, a member-permeable protein, PTEN-Long can act on the PI3K-AKT-mTOR pathway and enter neighboring cells following its secretion from cells, dephosphorylating PIP3, antagonizing PI3K-AKT signaling and inducing cell death in renal cell carcinoma (18). In the present study, we demonstrated that protein expression of PTEN-Long was reduced or completely lost in liver cancer patients at high frequency, suggesting that it plays an essential role in liver cancer via PTEN-Long-PI3K-AKT pathway through inhibiting tumor cell proliferation, migration and inducing apoptosis and autophagy. These effects are similar to the significant role of PTEN in the development of liver cancer.

The PI3K/AKT/mTOR pathway is involved in tumor formation, cell cycle progression, cell cycle progression, survival, and even apoptosis. Apoptosis occurs by two pathways, the “exogenous” and “endogenous” pathways mediated by death receptors and mitochondria, respectively. These pathways clear damaged or redundant cells, indicating it as an essential target of cancer (27). These two pathways are related to the Bcl-2 family and mitochondrial proteins (28); Bcl-2 is usually maintained by PI3K activity in the cytoplasm, where it regulates the degree of Bax translocation to mitochondria (29). The PI3K/AKT/mTOR pathway is a modulator of autophagy (30). In the present study, we observed that treatment with purified PTEN-Long suppressed levels of p-AKT, p-PRAS40, and p-mTOR with upregulated expression of Bax and the activity of apoptosis-related proteins in HepG2 cells. PTEN-Long downregulated expression of Bcl-2, but also changed expression of autophagy-related proteins (p62, beclin-1, and LC3BII/I), suggesting that apoptosis induction and autophagy may be mediated by suppression of the PI3K/AKT/mTOR pathway. More importantly, exogenously added PTEN-Long to HepG2 cells resulted in reduced p-AKT, p-PRAS40, and p-mTOR, which inhibited tumor cell proliferation and migration without any significant change induced in HepG2 cells after treatment with exogenous PTEN protein. In summary, our findings suggest that PTEN-Long participates in the development of liver cancer, and it might serve as a functional tumor suppressor protein.

## Data Availability Statement

We have approved the statement that the data presented in this article is publicly available, and further queries can be directed to the corresponding author/s.

## Ethics Statement

Ethical approval was not provided for this study on human participants because the Ethics Committee had been not established in that time. The patients/participants provided their written informed consent to participate in this study. Written informed consent was obtained from the individual(s) for the publication of any potentially identifiable images or data included in this article.

## Author Contributions

HL and LT: conception and design. HL: administrative support. SZ and JZ: provision of study material or patients. LT and ZX: collection and assembly of data. QM and LT: data analysis and interpretation. All authors wrote the manuscript and approved the final version of the manuscript.

## Funding

This study was supported by Advanced Key Scientific and Technological Programs of Ningbo, Grant Number 2013C51009, Public Technology Application Research Project of Zhejiang, Grant Number 2017C35002, Project of Zhejiang Medical and Health Platform Plan, Grant Numbers 2016DTA009 and 2019ZD046, Project of Zhejiang Medical Technology, Grant Number 2016KYB307, Ningbo Natural Science Funding, Grant Number 2018A610376, and Ningbo Clinical Medical Research Center, Grant Number 2019A21003.

## Conflict of Interest

The authors declare that the research was conducted in the absence of any commercial or financial relationships that could be construed as a potential conflict of interest.

## Publisher's Note

All claims expressed in this article are solely those of the authors and do not necessarily represent those of their affiliated organizations, or those of the publisher, the editors and the reviewers. Any product that may be evaluated in this article, or claim that may be made by its manufacturer, is not guaranteed or endorsed by the publisher.
