# Corrigendum: Purified PTEN-Long induces liver cancer cells to undergo autophagy and apoptosis

**DOI:** 10.3389/fsurg.2022.982545

**Published:** 2022-08-03

**Authors:** Lin Tan, Zeping Xu, Qiqi Mao, Shaocheng Zhou, Jie Zhu, Xie Zhang, Hong Li

**Affiliations:** ^1^ Department of Hepatobiliary Surgery, The Affiliated Hospital of Ningbo University, LiHuiLi Hospital, Ningbo, China; ^2^ Department of Gastrointestinal Surgery, The Affiliated Hospital of Ningbo university, Ningbo First Hospital, Ningbo, China; ^3^ Department of Pharmacy, The Affiliated Hospital of Ningbo university, LiHuiLi Hospital, Ningbo, China

**Keywords:** PTEN-Long, autophagy, apoptosis, liver, cancer

A corrigendum on Purified PTEN-Long induces liver cancer cells to undergo autophagy and apoptosis By Tan L, Xu Z, Mao Q, Zhou SC, Zhu J, Zhang X, Li H. (2022). Front. Surg. 9:767611. doi: 10.3389/fsurg.2022.767611

**Order of correspondence should be changed to (reversed order):**

Hong Li

*lancet2017@163.com*

Xie Zhang

*rennie22@126.com*

**Incorrect affiliations:Instead of**

Lin Tan^1†^, Zeping Xu^2†^, Qiqi Mao^3^, Shaocheng Zhou^3^, Jie Zhu^3^, Xie Zhang^2*^ and Hong Li^3*^

^1^Department of Gastrointestinal Surgery, Ningbo First Hospital, Ningbo, China, ^2^Department of Pharmacy, The Affiliated Hospital of Ningbo University, LiHuiLi Hospital, Ningbo, China, ^3^Department of Hepatobiliary Surgery, The Affiliated Hospital of Ningbo University, LiHuiLi Hospital, Ningbo, China

**It should be:**

Lin Tan^1,2†^, Zeping Xu^3†^, Qiqi Mao^1^, Shaocheng Zhou^1^, Jie Zhu^1^, Xie Zhang^3*^ and Hong Li^1*^

^1^Department of Hepatobiliary Surgery, The affiliated hospital of Ningbo university, LiHuiLi Hospital, Ningbo China ^2^Department of Gastrointestinal Surgery, The affiliated hospital of Ningbo university, Ningbo First Hospital, Ningbo, China; ^3^Department of Pharmacy, The affiliated hospital of Ningbouniversity, LiHuiLi Hospital, Ningbo, China ^†^These authors have contributed equally to this work

**Incorrect Funding**

In the published article, there was an error in the Funding statement. The original read: “The funding statement for Advanced Key Scientific and Technological Programs of Ningbo, Grant Numbers 2013C51009 and 2018A610376”, “Ningbo Natural Science Funding, Grant Number 018A610376”] were displayed in original text.

**The correct Funding statement appears below:**

This study was supported by Advanced Key Scientific and Technological Programs of Ningbo, grant number 2013C51009; Ningbo Natural Science Funding, grant number 2018A610376.

The authors apologize for this error and state that this does not change the scientific conclusions of the article in any way. The original article has been updated.

